# Generational Diversity in the Workplace: Psychological Empowerment and Flexibility in Spanish Companies

**DOI:** 10.3389/fpsyg.2019.01953

**Published:** 2019-08-23

**Authors:** Ignacio Sobrino-De Toro, Jesús Labrador-Fernández, Víctor L. De Nicolás

**Affiliations:** ^1^Facultad de Ciencias Económicas y Empresariales, ICADE, Universidad Pontificia Comillas, Madrid, Spain; ^2^Facultad de Ciencias Humanas y Sociales, CHS, Universidad Pontificia Comillas, Madrid, Spain

**Keywords:** psychological flexibility, psychological empowerment, generation, millennial, diversity

## Abstract

Intergenerational diversity is a universal fact in sustainability and today’s work environment. Current studies seek to find differences that exist between these generational groups that coexist, cooperate, and sometimes compete in business organizations. Sixteen focus groups have taken place, four for each generation to find the differences that may exist depending on that group membership. Specifically, the psychological empowerment and psychological flexibility variables have been analyzed, which have already shown their relevance to improve performance. Results show differences between the older generations (BB and Gen X) and the younger ones (Gen Y and Gen Z).

## Introduction

The development of the Internet and data analysis (Geczy et al., 2014), the abundance of information (Southwell, 2005), the globalization (Mark, 1996), the growing interest in diversity (Guajardo, 2014), the increased consumer power (Kucuk, 2008), or what is known as the sharing economy (Belk, 2018), all represent deep changes which are affecting people and organizations to a great extent. This environment is now defined as VUCA (Whiteman, 1998), an acronym of Volatility, Uncertainty, Complexity, and Ambiguity.

Companies are responding to this new environment in very different ways. One of the most common is the intensification of work, which is understood both as the hours worked as well as the intensity of the work. This intensification is reaching the acceptable limits (Brown, 2012) and at the same time has resulted in pressure on employees moving from peaks and troughs to becoming something continuous. This has associated implications both for people and companies (Dawson et al., 2001).

At the same time, employees’ commitment levels are at very low levels. As a result, only 13% of employees say that they are committed to their company (Gallup, 2013). This requires greater attention if we remember the direct link between commitment and performance, a link which has been widely demonstrated (Harter et al., 2002).

The Human Resources function therefore has many aspects to manage which were not present in past decades. In a survey from 2013 carried out among 1,300 Human Resources professionals, 70% said they could not deal with complexity, with 60% saying they had serious doubts about their organization’s ability to deal with this increasing complexity (Lumesse, 2013).

Given that the ability to adapt is essential in order to achieve good results (Heugens and Lander, 2009; Reeves and Deimler, 2011), people management in organizations needs to adopt new tools and/or review existing ones in order to continue adding value to organizations according to this new VUCA environment. In modern organizations, we may find employees of four different generations. Generational diversity is essential to face the volatility and uncertainty but at the same time it may increase complexity regarding people management (Amayah and Gedro, 2014). A better understanding of this generational diversity will help to orientate politics and human resources practices.

Within this review of existing tools, we have identified two which have a significant impact with regard to performance and helping people to adapt to their professional environment: psychological empowerment and psychological flexibility. Up to date, there are no studies that analyze these concepts with the generational aspect of the employees. This study seeks to strengthen our understanding of these topics while identifying possible differences by analyzing them from a generational perspective, knowing that the diversity of human capital is present in modern organizations (Shen et al., 2009; Page, 2010).

### Generation, an Ambiguous Concept

Generational differences in the workplace as a research and intervention topic have recently grown significantly in popularity (Joshi et al., 2011; Lyons et al., 2015; Campbell et al., 2017). The number of widely circulated articles, media reports, and blogs has grown even more significantly too. At the same time, in the management world, there are numerous human resources consulting initiatives which consider intergenerational diversity and intervention policies are being created based on these.

Karl Mannheim, a pioneer in the conceptualization of the term generation, proposed that a generation, any generation, is determined by participation in the same events. These events are the source of vital contents that are fixed in the consciences of people as the “natural” way in which the world exists. As a result, a natural image of the world is formed which guides others, is the base from which subsequent events are understood; it is the code for interpreting everything that happens. For Mannheim (1993), the process is very determinant because it happens in the first stage of life. The active participation in the social currents that constitute and give meaning to the historical moment creates the generational bond. This is how one generation creates a new historical situation (Mannheim, 1993; Edmunds and Turner, 2005).

Growing in a group does not only involve making assessments based on these interpretation principles which the group are characterized by, it also involves capturing certain aspects, those nuances, and meanings of certain concepts in which reality is present within the group (France and Roberts, 2015). The individuals are linked through a generational connection, only to the extent that they participate in social events which represent and give meaning to the respective historical moment, and to the extent that they take part (both actively and passively) in new interactions which make up the new situation (Mannheim, 1993; Pilcher, 1994).

To define and identify this great complexity with the date of birth is a great simplification (Dimock, 2019). This limitation does not prevent the occurrence of many and very diverse investigations in which the date of birth has been used as a key criterion of differentiation (Kowske et al., 2010; Andert, 2011; Suomäki et al., 2019).

It is easy to think that, if someone has grown up and developed in a different world to someone else in history, they might have different ways of thinking, even if they are from the same place. In the academic and empirical studies environment, there is some controversy surrounding the suitability of the “generation” concept, its explanatory characteristic, and its reliability and applicability. The fundamental reproaches to these studies relate to the explanatory weakness of the generation concept (Giancola, 2006; Ng and Feldman, 2010; Constanza et al., 2012; Constanza and Finkelstein, 2015). Similarly, and equally as important, is the intrinsic link between the generation concept and other variables such as age, historical period, and cohort when it comes to belonging to a group (Campbell and Twenge, 2014; Segers et al., 2014), which according to these criticisms make this an ambiguous concept.

On the other hand, it is recognized as an area of research which lacks maturity and empirical contrast, although it is growing and slowly consolidating (Lyons and Kuron, 2014).

There are studies that talk about differences in generations, for example, Twenge and Campbell (2008), show how generation Y (Gen Y) has higher levels of self-esteem, anxiety, and narcissism. On the other hand, other studies show that there are practically no differences between generations (Hart et al., 2003), Korn (2010) concludes that at the organizational level the differences between generations are not very significant (Korn, 2010).

It is important to mention that one of the areas where this increase is most evident is in the study of how the differences in generational identity have consequences in the workplace. From the initial studies focused on the concept of generational identity itself (Dencker et al., 2008; Joshi et al., 2010), there has been a slow but steady increase and deepening in the consequences of values at work, motivation, and other variables relating to workplace performance (Twenge et al., 2010; Sakdiyakorn and Wattanacharoensil, 2017).

Until very recently, bureaucratic organizations had a holistic culture in which habits and ways of working were created and determined, and these concealed diversity as well as the novelty of new agents or employees (Lok and Crawford, 2004). These days, although these socialization phenomena are still present in company culture, they are no longer so prevalent; autonomy and self-expression are considered essential for workers’ knowledge (Robbins and Judge, 2009).

### Employees’ Psychological Empowerment

The concept of empowerment (applied in companies), started to become relevant when Conger and Kanungo (1988) identified it as a key component for organizational management and effectiveness, defining it as “a motivational construct aimed at enablement rather than delegation”. Kanter (1993) considered empowerment as the mobilization of resources, information, and support to get things done, incorporating the concept of reporting lines, both formal and informal.

There are two different interpretations of empowerment in the literature, the first of which is known as structural, based on resources and the organization’s ability to act with regard to its workers (MacDuffie, 1995; Wright et al., 2003; Gibson et al., 2007). The second interpretation of empowerment is linked to intrinsic motivation as well as employees’ reaction to resources, information, and support which are made available (Spreitzer, 1995). This interpretation is more closely linked to the beliefs of the employees themselves (Harrim and Alkshali, 2008), and is known as psychological empowerment.

Thomas and Velthouse (1990) defined psychological empowerment as being formed of four aspects: meaningfulness, competence, choice, and impact. Based on this theoretical model, Spreitzer (1995) created a measurement scale, substituting “meaningfulness” with “meaning” and “choice” with “self-determination” (Liden et al., 2000). Spreitzer’s (1995) model provides psychological empowerment with a motivational dimension; that is, people who are empowered should demonstrate an active attitude toward work, incorporating their own beliefs to their role within the organization (Fernández et al., 2015).

These four factors can be seen as a description of the relationship between the employee and their work. Therefore, competence considers the relationship between the person and the tasks they carry out; meaning describes the link between the employee’s objectives and goals with those of the organization. Self-determination describes the freedom with which the employee carries out tasks and the relationship with the organization’s rules. Finally, impact reflects the perception that the employee has with regard to the results of their performance.

In recent decades, psychological empowerment has been widely used in studies on workplace characteristics (Aryee and Chen, 2006; Chen et al., 2007); a strong link between intrinsic motivation and creativity (Zhang and Bartol, 2010), supervision and leadership styles (Kim and Kim, 2013) was identified. Relationships between this variable and results in the workplace have also been identified, with negative impacts on employee turnover being identified (Kim and Fernandez, 2017) and positive impacts between empowerment and workplace satisfaction (Koberg et al., 1999; Liden et al., 2000; Carless, 2004; Aryee and Chen, 2006), with the level of commitment and improvement in the company’s performance (Sahoo et al., 2010; Yao et al., 2013).

Although psychological empowerment has been widely investigated, there are no studies that relate it with the generations which would help to better orientate HR policies and practices.

### Psychological Flexibility

Psychological flexibility is the objective of clinical intervention known as Acceptance and Commitment Therapy (ACT). As a result, it is the final outcome of a process in which a number of psychological variables (and their evolution) are taken into account.

ACT is a therapy based on Relational Frame Theory, which facilitates a change in behavior based on the way that people establish relationships between words and events (Hayes et al., 2001). As well as cognitive and behavioral aspects, ACT also introduces a more transcendent component with elements such as values. Its objective is to introduce greater flexibility in terms of cognition, helping the person to confront situations from a different perspective, allowing the person to establish a new Relational Frame (Relational Frame Theory), and as a result, new behavior (Hayes, 2004).

ACT is present across different types of intervention among which the following can be highlighted: practicing mindfulness, the use of metaphors, personal experience processes, learning linked to the definition and achievement of goals and objectives, identification of values, etc. (Hayes et al., 2006).

ACT has been shown to be hugely effective in helping people tackle complex situations such as anxiety, stress, depression, psychosis, addictions, acute pain, etc., and has also proven highly effective in reducing and transforming negative thoughts (Zettle and Hayes, 1986; Bach and Hayes, 2002; Ruiz, 2010, 2012; Jansen et al., 2017). In summary, ACT is a collection of tools which are proven to be effective in helping people change their thoughts and behavior, even with complex problems.

**Figure 1:**
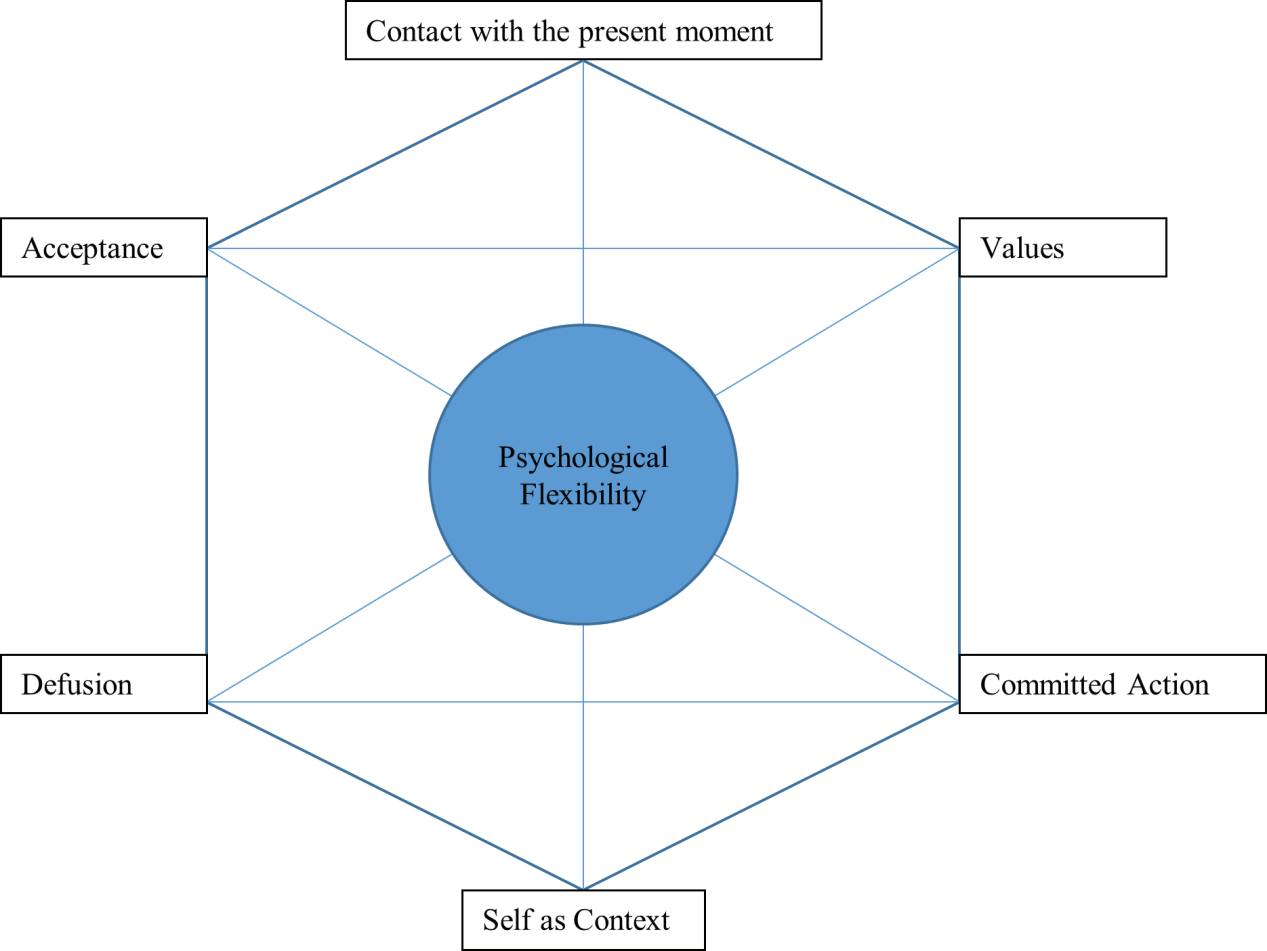
Prepared by the authors based on Hayes et al. (2006), p. 25.

This therapeutic approach is based on a series of components which are essential for understanding and achieving psychological flexibility. According to Hayes (2004), who created this approach, there are six: contact with the present moment, values, committed action, self as context, defusion, and acceptance (Hayes et al., 2006). These six elements revolve around two poles: awareness and acceptance, and commitment and adopting new behavior (Hayes et al., 2006). The six elements mentioned are presented in a hexagon known as the “hexaflex” (Hayes et al., 2006), as shown in Figure 1.

The aim of ACT is to help individuals to be in touch with, embrace, and evaluate their current circumstances in order to act in a better way in various situations (Bond et al., 2006). This means being psychologically flexible. We understand psychological flexibility as the ability to connect with the present moment, with an attitude that embraces whatever is happening in the moment, and as a result of this acceptance, acting with awareness and consistently based on the person’s own values (Hayes et al., 2004a,b). It is very closely linked to feeling like a protagonist rather than a victim, as well as the ability to choose and keep up the pace to achieve the end result, despite any difficulties that may be encountered on the way.

One of the areas in which human beings confront situations where their psychological flexibility is put into practice is the workplace. There have been many empirical studies that have explored psychological flexibility in the workplace, more specifically with regard to health in the workplace (Flaxman and Bond, 2010; Lloyd et al., 2013).

Multiple longitudinal studies have shown that there is a correlation between higher levels of psychological flexibility, and work related results, including better productivity, improved mental health, and increased ability to learn new skills at work (Bond and Bunce, 2003; Bond and Flaxman, 2006; Bond et al., 2016). It has also been found that people with higher levels of psychological flexibility make better use of the resources available to them in the work environment. Bond et al. (2008) demonstrate that the highest levels of psychological flexibility improved the positive impact of a job role redesign. Although all these investigations indicate that psychological flexibility may help organizations to help people to adapt to new changes, there is no information about the differences in psychological flexibility trough generations. This knowledge would help to be more effective in HR actions and facilitate company’s adaption to environment challenges.

### Objective of the Research

The investigation tries to increase the current knowledge of the generational diversity within the professional environment to help Human Resources areas to orientate their practices. In a more specific sense, this research is to try to better understand two variables which have an important impact on helping workers to adapt to an ever-changing environment. Therefore, we will analyze these based on a third component: generational diversity. This research aims to answer the question of whether there are differences in the aforementioned discourse depending on the generational group, in relation to their psychological empowerment and psychological flexibility at work.

Our initial hypothesis is that there may be differences in both psychological variables due to being from a different generation. Those generations with more experience and more opportunities to reflect on their experiences show greater levels of flexibility, and those groups with more professional experience and a greater sense of their role in the company also show clear differences with regard to psychological empowerment.

## Methodology

This is a qualitative study based on focus groups. These focus groups have been conducted by a model and a method with the aim of discussing and concluding the objectives of the research.

### Focus Groups

All participants were volunteers. They were selected by their managers and HR Directors looking for diversity in educational level, years in the company, sex, and hierarchical level. In total, 16 focus groups took place, four for each age group that was being studied; 156 workers participated in this stage of the research, of which 88 were male and 68 were female.

The research team is incredibly grateful to the companies who provided these employees: Baxter, BBVA, Enagás, Ferrovial, Gas Natural Fenosa, Heineken, Mapfre, Meta4, Orange, Sabadell, Sandoz, Santander, Pascual Hermanos, REPSOL, and Universia. These companies are leaders in their sectors, and represents baking, energy, construction, consumer goods, and pharma industries. All the groups were recorded, and these recordings were transcribed in order to analyze the discussion. As a result of these groups, a “content base” was created to hold all the information collected during the discussions.

Throughout the process, ethical standards were respected according to the Helsinki Declaration (World Medical Association, 2001). All participants gave their written informed consent to be recorded and to use the information extracted from the groups. There was complete transparency with the participants.

As previously said, the concept of generation includes historical, social, and psychological variables. It is a concept with multiple faces and related to each other with great complexity, setting the limits of that complexity between two birth dates is a simplification.

The generational dimension which this intergenerational study hoped to provide presented various challenges due to the various grouping options and the lack of clear consensus defining each generation. Based on the meta-analysis by Constanza et al. (2012), the team decided to define the following four groups, according to their year of birth: Baby Boomer – BB (1955–1969), Generation X – Gen X (1970–1981), Generation Y or Millennials (1982–1992), and lastly Generation Z – Gen Z, those born after 1993^1^.

Their availability to attend the group meetings was also taken into account. This simplified and arbitrary way of defining a generation has been widely criticized (Constanza et al., 2012; Constanza and Finkelstein, 2015), and the need to carry out a deeper analysis on the variables involved in the generation concept has been emphasized, so more than just the date of birth is considered (Lyons and Kuron, 2014; Wang and Peng, 2015). Lyons and Schweitzer (2017) adopt a more comprehensive approach, based on the phenomena of social categorization and identity (Lyons et al., 2015).

In all the focus groups in which people had been categorized as members of a generation, there was discussion among the group in terms of their awareness of belonging to that group and how that categorization fits with their own perceptions. The aim of this article is not to review the components of social categorization, but we should highlight that only two of the participants across all the groups were uncomfortable with this categorization and identified themselves as belonging to a different category. The rest were satisfied with the proposed examples, which is much higher than in previous studies (Roberto and Biggan, 2014; Lyons and Schweitzer, 2017).

The four groups from the BB generation took place between March 2016 and January 2017, with a total of 36 people taking part, of which 22 were women and 14 were men. The groups were made up of five, nine, 11, and 11 people. The four Gen X groups took place between February 2016 and September 2016. In total, 41 people took part, of which 19 were women and 22 were men. The groups were made up of 15, seven, eight, and 11 people in each. The four Gen Y groups took place between March 2016 and May 2016 with 43 people taking part. There were 22 women and 21 men, and each group was made up of 12, 11, seven, and 13 people. Gen Z was studied between May 2016 and March 2017, with a total of 36 people taking part (25 women and 11 men). Four groups took place with six, eight, 10, and 11 people.

All the participants were current employees or interns. Interns were included because of the young age of the last generation represented (younger than 23 years old), of the companies that provided samples the number of under 23 s was negligible. Interns were included and, although they do not have permanent employment with the company, it is the only opportunity to see how members of this youngest generation are adapting to the workplace. In addition, interns represent many of the other employees’ discourses. It is common for these interns to be recognized as the main source of young talent and a “breath of fresh air” in the company.

It is also necessary to mention that from this generation there has also been access to young people who are “enjoying” a graduate program, something which demonstrates exceptional initiative, preparation and ability. In either case, the representatives of Gen Z which we have had access to (interns, employees, or graduates), are not the typical example of this generation; rather they are at the cutting edge.

### Model and Method

Both psychological empowerment and psychological flexibility have been studied quantitatively using scales. The Psychological Empowerment scale, known as the “Psychological Empowerment Instrument” was created by Spreitzer (1995), and consists of 12 items divided into four factors, with each of these made up of three items. The original scale for measuring psychological flexibility was created by Hayes et al. (2004a,b) and consists of seven items. Subsequently, Bond et al. (2011) created the AAQ – II. Finally, Bond et al. (2013) created the WAAQ adaptation of the scale in a professional context.

However, this study does not aim to measure but rather better understand the generational component of each concept relating to current employees who are experiencing the pressures of a job market full of uncertainty and volatility. We were interested to understand perceptions of key aspects in their environment, both of themselves and of the possibilities within the world of work.

The focus groups were between one hour and an hour and a half long. They were led by the research team and were always organized around three key factors, which we could say are existential.

**Figure 2:**
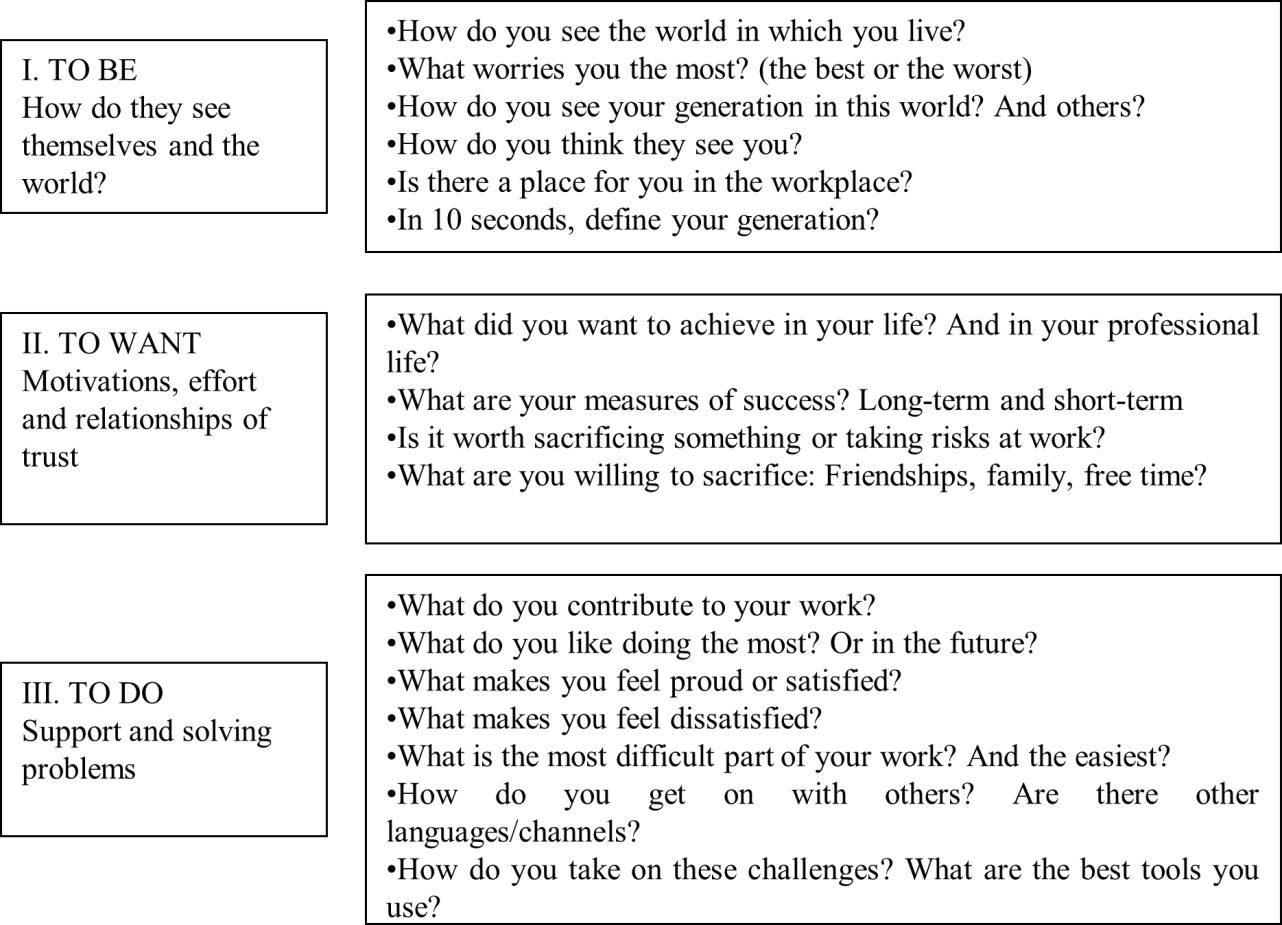
Prepared by the authors. Examples of the questions asked to the employees in the focus groups.

Figure 2 shows the general framework which all the focus groups were based on. The questions are illustrative; the aim was for the discussion in the group to flow naturally, while facilitating spontaneous access to the topics based on an open and trusting environment. All the groups did start with the same question: “How do you see the world in which you live in?” The moderator was responsible for facilitating the discussion, encouraging members to speak, asking overly talkative members to let others speak and encouraging all members to participate. In addition, the moderator was responsible for taking notes that may led to emerging questions. In this case, the moderator also presented to the participants of the focus group the questions that are shown in Figure 2, only when it was necessary. In many cases, the group itself was generating the discourse (Onwuegbuzie et al., 2009).

The objective is to be able to analyze the consistency of the discourse, as well as identify elements of psychological empowerment and flexibility, based on the detailed discussion on the realities faced in the workplace, avoiding the more typical questions on empowerment and flexibility so as not to steer the participants and skew the results.

The analysis of the employees’ discussion content started with the creation of an initial matrix which uses all the concepts such as empowerment (meaning, competence, impact, and self-determination), as well as psychological flexibility (connection with the present, expressed values, committed actions, cognitive defusion, acceptance, self as context), separating self-attributions from external ones. This first classification filter was organized both by individual or personal self-attributions as well as groups or generations, and the same for the external attributions.

The research team adopted a form of discourse analysis inspired by Wetherell and Potter (1988) and Klevan et al. (2018). Although presented as a step-by-step description, a strict sequence has not been followed. The identification of possible discourses in the text and how they are featured are better understood as constructs resulting from the back-and-forth movements between the steps, which were as follows:

(1)Read the text repeatedly to become familiar with the data.
(2)Coding of the sections in the material, focusing on the content of possible discourses and how they were expressed.
(3)organize the coded material into clusters according to the content and the way in which it was expressed.
(4)Organize the content clusters in possible discourses and finally.
(5)Question possible discourses in relation to each focus group with all the data as a whole, looking for possible patterns in terms of variations and consistency.

Following this, the data were summarized (separating units, grouping, and classifying elements), arranged, and transformed. Based on this initial transformation of the text corpus, an analysis was carried out in various stages of recurrent open coding for each category, in a continuous coding and categorization process in order to facilitate comprehensive analysis of the recurrent elements, the responses are organized and grouped into emergent categories.

## Results and Discussion

The study carried out among these 16 focus groups brings into question the existence of significant differences between the four generations. The discussions gain differential consistency by being separated into two groups. During the analysis of the texts, it has been demonstrated that separation among youngsters, with little work experience (born after 1982, Gen Y and Gen Z) and older people, with greater experience and who have been working longer (born before 1982, BB and Gen X) generates greater and clearer variability between groups. Based on the data collected, it seems that differences are potentially related to the amount of personal and professional experiences that older people accumulate.

It is evident that in these two groups, the most extreme generations (older people about to retire – BB and young interns still in education – Gen Z) have a certain ability to be differentiated, and in some cases, it is possible to see some differences, although very specific between the four generations. In either case, it is necessary to highlight that a single characteristic has not been identified that is unique to each generation, and as with many other differential aspects, the variability within the group is greater than the variability between groups.

It is obvious that due to the simple fact of making this social categorization and activating it in terms of creating the focus groups (four in each of the generations), there is almost instantly homogenization with the group and differentiation between groups.

In addition, most groups manifested that the focus groups had contributed to increase their awareness of themselves as members of a specific generation and their capacity to influence in their jobs (psychological empowerment) and in their lives (psychological flexibility).

### Psychological Empowerment

All the groups, regardless of their age or experience, have a good perception of themselves in terms of the relation between their competences and the work they carry out. In general, they see themselves as having the power and ability to instigate effective change in the world they have chosen, especially with regard to the meaning they give to their career path and the perception of their own competences. In both dimensions (competence and meaning), the discussions are truly positive.

It is important to mention that there is also a more negative discussion with regard to the lack of control and lack of awareness for the meaning of life, but it is a minority and not exclusive to any of the generational groups.

The meaning of life and work for older people (BB and Gen X) is based on their sense of responsability for what is going on in the world, as well as on what is going on in their workplace and home. They are people who feel and express the weight of responsibility over others, whether they are colleagues or children. In some instances, during the discussions, a sense of urgency is even detected with regard to the opportunity of improving things. They live and feel with free reign and they are the ones who have this meaning of life.

The youngest group (Gen Y and Gen Z) is very different. An idea that has been expressed frequently in the groups is that they have been charged with being the leaders of change. The purpose of their work is to change things, transform, and make all these bureaucratic, administrative and hierarchical processes more effective as they are making decision-making too slow. The objective of their work is to transform it, not only to improve it but also to make it fun and motivating.

Gen Y and Gen Z clearly identify as having less impact and being less capable of self-determination. This frequently manifests itself as a complaint, highlighting the obstacles they face in terms of empowerment, and also showing the contradictory nature of the “official discourse” on the importance of young talent, who also continuously face endless challenges emanating from a hierarchy they consider to be obsolete and out of place. In other cases, they do this by accepting they have less experience and therefore realize there is a need to have challenges and leaders who help them to improve their skills and power.

The approach that BB and Gen X take in terms of their impact and self-determination is much more active and satisfactory. They use more tools, skills, and capabilities, which helps to put them in a position of responsibility. In this sense, among these older people (who have a greater sense of perspective), it is more common for them to reflect on the relevance of their contributions and the ultimate impact they have had.

### Psychological Flexibility

Multiple references to psychological flexibility variables have been found, although there is no clear differentiating discourse in an age group. It should be mentioned that by merely participating in the focus groups, this put our participants in a position where they “objectified their subjectivity” through the contrast in dialogue. This is an exercise (albeit one-off and planned), which Hayes et al. (2006) call “self as context.”. There were many diverse individual contributions, although no generational differences were found.

It was clear that, among all the generations, people were becoming aware of the job market conditions in Spain, although the way in which they approach this was as diverse as the people who made up the groups themselves.

The level of psychological flexibility among the participants across all generations can be improved. In all the groups, there is a lack of awareness in terms of being able to manage private events, a task which is difficult for everyone in this volatile and complex environment, something which all of the generations complain about. All the generations (including the youngest Gen Y and Gen Z) admit that they find the current uncertainty very challenging.

The biggest difference between the discussions took place again between BB and Gen X and Gen Y and Gen Z (younger people). BB and Gen X feel the need of taking charge of their lives, while for Gen Y and Gen Z, most of the discussion related to them being victims of a situation and a reality which moves them from one place to another and determines their current status.

The youngest generation, known as Gen Z, are the ones who most describe a situation linked to a crisis which defines them. This vital crisis or economic depression situation governs them and affects them even if they know they are very well prepared.

It was also seen among these youngsters (Gen Z) that they have had great success entering the job market, they are very critical and negative in terms of the learning and work environment they are experiencing, in which only their ability to innovate and distance themselves from situations will lead to success. This discourse on innovation and the autonomous search for resources was raised by a minority, and we understand that it has appeared as a result of having access to a sample of people who, by their special characteristics, have stood out and integrated into the job market successfully early on. Many of them even mentioned friends and family who had not as much “luck.” The general sentiment is that of complaint and regret, without delving any deeper.

Another difference which is evident, and which differentiates the two younger generations, is that in Gen Z there is a greater hunger for success and achievement, as well as more initiatives for developing alternative plans. They have seen how their older brothers and uncles, who despite having university degrees, have not been able to enter the job market, and as a result, they have always considered university to be insufficient and have sought complementary training.

In terms of accepting and confronting events faced by both young (Gen Y and Gen Z) and old (BB and Gen X), we can see differences which are clearly linked to people’s baggage and past experience. In general, there is a greater sense of accepting and confronting private events among people from BB and Gen X, without a doubt it is this experience which has taught them that it is better to take these on and confront them rather than avoid them. Gen Y and Gen Z see themselves as having more tools for avoiding these, and they even consider avoidance as being easier and more convenient due to the opportunities provided by new technology and networks. Due to the functional ubiquity of mobile devices, these youngsters have the option to never close down a line of action; they are involved in everything without giving up on anything, which seems like a way of avoiding confrontation. They complain that they do not have enough time or opportunities to deal with events in a reflective and profound manner.

It is important to understand that the young population (Gen Y and Gen Z) is entering the job market or has only recently entered. Furthermore, it is an extremely unstable and volatile market; the conditions are unfavorable for having an adequate self-perception within the context or associated defusion. They feel change and uncertainty.

There is also a difference again between BB and Gen X and Gen Y and Gen Z when it comes to articulating a coherent support between values and actions, something which is much more prevalent among older people (BB and Gen X), it seems that it is necessary to have a history of experiences which provide opportunities to reflect on the coherence and consistency between value and action. These experiences and learnings are evident among the older participants during the discussions and they are linked to values such as loyalty, commitment, and doing things properly.

## Conclusion

Generational differences in the workplace have become a widely discussed topic in multiple publications in recent years, and there have also been countless experiences in human resources departments. It is also true that there is an open discussion on the suitability of this segmentation by generation (Constanza et al., 2012; Lyons and Kuron, 2014). There are doubts as to whether this segmentation is explanatory or a significant enough source of behavioral diversity. It is not easy to distinguish the generational effects with the effects produced by age, maturity, and experience (Twenge, 2000; Macky et al., 2008).

In this study, we have stated that it is these developmental elements which form the basis of the different discourses which have been expressed.

No differences have been found between the four proposed age groups, although clear differences have been found in the discussions with regard to psychological empowerment and psychological flexibility among employees born before 1982 (who as a result have more work and life experiences as is the case of BB and Gen X) and younger people who have few years of professional experience (Gen Y and Gen Z).

In terms of empowerment, both groups showed a positive self-image, although their empowerment was qualitatively different. Therefore, the role of their work within the wider population is determined by their responsibility for others and their work, and this responsibility has a sense of urgency. Among the younger population, work is important for achieving transformation and a different future.

Gen Y and Gen Z from our sample complain about the lack of self-determination as they consider themselves to be constrained by older people’s authority and the rules of bureaucratic structures, which they criticize heavily.

The differences in psychological flexibility are visible between older people (BB and Gen X) and younger people (Gen Y and Gen Z) who avoid confrontation, especially when it comes to interpersonal conflicts and giving up or not finding alternatives during decision-making. Therefore, youngsters have a greater ability for cognitive fusion between their thoughts and the reality in which they live, and they often feel like the victims.

Generational replacement is not a trivial topic in societies and organizations. Knowledge transfer is essential in order to secure and grow companies, and these should ensure that it takes place.

The focus groups carried out in this study have not shown clear differences between the four proposed generations, although there are many common themes as they all share the same cultural, economic and organizational situation. There have been more significant similarities and agreements than there have been differences. In many cases, these differences are a result of stereotypes which are more or less appropriate which have left a mark on society, and which tend to stereotype; as soon as the discussions became a bit longer and deeper, the differences once again become evident. There is, as has always been the case, a tension between the groups and people with experience (BB and Gen X) and those who want to get experience quickly (Gen Y and Gen Z). These two groups (young people and old people) have always existed and, although there are clear differences between them, the knowledge transfer between them remains as present as always, with the exception that in these “millennial times” this transfer is especially difficult and pressing.

The understanding of all these differences, based on age, may help companies to better use the psychological empowerment and psychological flexibility initiatives in order to facilitate the adaptation to the current VUCA environment. This understanding will be able to illuminate future strategic actions for Human Resources departments when facing the generational diversity challenges.

## Data Availability

All datasets generated for this study are included in the manuscript and/or the supplementary files.

## Ethics Statement

Ethical review process is not required as per the Spanish Law of Biomedical research 14/2007, July 3 since this is not a biomedical and clinic research. This study does not develop any clinical trials and does not involve patients; therefore, no written informed consents of the patients are required. This study is a qualitative research based on interviews and focus groups. The participants in these groups were informed according to the Spanish Law 5/1992, and all the information recorded in these groups was treated by anonymous form according to the previous referred law applicable to Spanish Universities. This study does not involve animal subjects.

## Author Contributions

IS-D contributed to idea, redaction, and interviews. JL-F contributed to redaction, assessment, and conclusion. VD contributed to review, recommendations, and bibliography information.

### Conflict of Interest Statement

The authors declare that the research was conducted in the absence of any commercial or financial relationships that could be construed as a potential conflict of interest.

## Footnotes

^1^In this article generations are named as BB, Gen X, Gen Y, and Gen Z.
